# Generation of Stilbene Glycoside with Promising Cell Rejuvenation Activity through Biotransformation by the Entomopathogenic Fungus *Beauveria bassiana*

**DOI:** 10.3390/biomedicines9050555

**Published:** 2021-05-17

**Authors:** Sang Keun Ha, Min Cheol Kang, Seulah Lee, Om Darlami, Dongyun Shin, Inwook Choi, Ki Hyun Kim, Sun Yeou Kim

**Affiliations:** 1 Division of Food Functionality Research, Korea Food Research Institute, Wanju 55365, Korea; skha@kfri.re.kr; 2 Divison of Food Biotechnology, University of Science and Technology, Daejeon 34113, Korea; 3 College of Pharmacy, Gachon University, 191 Hambakmoe-ro, Yeonsu-gu, Incheon 21936, Korea; kismc0511@naver.com (M.C.K.); darlami.om@gmail.com (O.D.); dyshin@gachon.ac.kr (D.S.); 4 School of Pharmacy, Sungkyunkwan University, Suwon 16419, Korea; seulah@kopri.re.kr; 5 Division of Life Sciences, Korea Polar Research Institute, KIOST, Incheon 21990, Korea

**Keywords:** *Beauveria bassiana*, resveratrol, biotransformation, microarray analysis, cell rejuvenation

## Abstract

A stilbene glycoside (resvebassianol A) (**1**) with a unique sugar unit, 4-*O*-methyl-D-glucopyranose, was identified through biotransformation of resveratrol (RSV) by the entomopathogenic fungus *Beauveria bassiana* to obtain a superior RSV metabolite with enhanced safety. Its structure, including its absolute configurations, was determined using spectroscopic data, HRESIMS, and chemical reactions. Microarray analysis showed that the expression levels of filaggrin, HAS2-AS1, and CERS3 were higher, while those of IL23A, IL1A, and CXCL8 were lower in the resvebassianol A-treated group than in the RSV-treated group, as confirmed by qRT-PCR. Compound **1** exhibited the same regenerative and anti-inflammatory effects as RSV with no cytotoxicity in skin keratinocytes and TNF-α/IFN-γ-stimulated HIEC-6 cells, suggesting that compound **1** is a safe and stable methylglycosylated RSV. Our findings suggest that our biotransformation method can be an efficient biosynthetic platform for producing a broad range of natural glycosides with enhanced safety.

## 1. Introduction

Resveratrol (RSV, 3,5,4′-trihydroxy-*trans*-stilbene), a natural compound commonly found in the skin of peanuts, grapes, raspberries, blueberries, and mulberries, is a physiologically active polyphenolic compound with potential antioxidant activity [1]. Naturally, RSV is present as a glycoside attached to glucose [2]. Many studies on RSV have demonstrated that it may play an important role in preventing and treating interactions involving cell signaling pathways and diseases associated with oxidative stress, inflammation, cancer, abnormal metabolism, and neurotoxicity [3].

To date, the clinical effects of RSV have not been sufficiently validated in cellular and animal studies. Additionally, there have been some limitations to its commercialization. For example, RSV is well absorbed in vivo, but it is rapidly metabolized to sulfo- or glucurono-conjugates [4]. RSV is safe at low doses, but it may have poor bioavailability in humans due to extensive hepatic metabolism [5]. Therefore, developing a drug for clinical use using RSV has been challenging. To overcome these problems, a new micronized RSV formulation—SRT 501—has been developed [6]. However, limitations associated with the metabolism and bioavailability of RSV continue to exist, and there is a need for RSV-derived compounds with enhanced bioavailability.

Natural product drug discovery includes the process of diverting more active molecules from the original active molecules of natural products, wherein a microbial transformation is a useful approach. The use of specific microorganisms that mimic mammalian metabolism to perform selective transformation reactions is advantageous due to the economically and ecologically friendly microbial transformations [7]. Attempts have been made to produce new RSV compounds using biotransformation and biosynthesis of RSV by microorganisms. A simple method to efficiently produce RSV from polydatin (piceid) using *Bacillus safensis* has been reported [8]. Owing to the practical challenges during the microbial production of RSV, some studies have been conducted on fungal strains such as *Botrytis cinerea*, which oxidizes RSV to produce RSV dimers such as restrytisols A, B, and C [9]. RSV 3-*O*-β-D-glucoside has also been produced from RSV using *Bacillus cereus*, also known as soil bacteria [10]. Additionally, the biotransformation technique using *Aspergillus* sp. yielded a new prenylated *trans*-RSV (arahypin-16) and RSV *trans*-dehydrodimer (leachinol F) [11]. However, their biological activities are not superior to those of RSV. In our preliminary experiments, biotransformation studies were performed using several dietary Lactobacillus and fungi. Interestingly, among them, it was confirmed that newly produced substances were detected only when the experiment was performed using *Beauveria bassiana*. Therefore, to obtain superior RSV metabolites with enhanced safety using microbial co-culture, we co-cultured RSV with tissue culture seedlings of *B. bassiana*, which is an important entomopathogenic fungus currently under development as a bio-control agent for various insect pests [12]. In the present study, resvebassianol A, a stilbene glycoside with a unique sugar unit, 4-*O*-methyl-D-glucopyranose, was isolated from the whole-cell fermentation of *B. bassiana*. The bio-functional superiority of the newly produced RSV metabolite, resvebassianol A, through biotransformation of RSV by *B. bassiana*, was further investigated using microarray analysis.

## 2. Materials and Methods

### 2.1. Microorganism

*Beauveria bassiana* (KCCM 60248) was purchased from the Korean Culture Center of Microorganisms (Seoul, Korea). All culture and biotransformation experiments were performed in potato dextrose agar (PDA, BD, Le Pont-de-Claix, France).

### 2.2. Metabolites of RSV Manufactured by B. bassiana

*B. bassiana* was incubated in PDA (0.4% potato starch, 2% dextrose, and 2% agar) at 26 °C for 3 days, and subsequently, the spores were collected from the plate surface using 0.85% saline and gauze filtration. The RSV metabolites were produced at different time points (0, 1, 3, and 7 days) at 26 °C for 72 h in a 100 rpm shaking incubator with initial inoculation concentrations of 5 × 107 spores/mL and RSV concentration of 100 µg/mL in the culture medium. The culture medium was treated with a two-fold volume of ACN, and it was vortexed, sonicated, and centrifuged at 8000 rpm for 15 min. The supernatants were filtered using a 0.2 µm syringe. The filtrate was concentrated and chromatographically analyzed using HPLC.

### 2.3. HPLC Analysis of RSV Metabolites

RSV metabolites were analyzed using a reverse-phase HPLC system (Waters Corp., Milford, MA, USA) with a photodiode array detector (model 2998, Waters Corp.) and a SunFire™ analytical C18 column (4.6 × 150 mm, 5 μm, Waters Corp.). The solvent system consisted of a gradient of solvent A (water:tetrahydrofuran:trifluoroacetic acid, 98:2:0.1, *v*/*v*/*v*) and solvent B (MeCN) with an initial composition of 83% A, isocratic to 75% A from 2 to 7 min, linear gradient to 65% A from 7 to 15 min, linear gradient to 50% A from 15 to 20 min, linear gradient to 20% A from 20 to 35 min, linear gradient to 83% A from 35 to 40 min, and linear gradient to 83% A from 40 to 45 min. Following this, the column was washed and reconditioned. The solution was eluted at a flow rate of 1 mL/min, and the UV spectra were monitored at 305 nm.

### 2.4. Isolation of RSV Metabolites

To optimize the incubation time, the culture medium was harvested after 1, 3, and 7 days, and the biotransformation yields were monitored at each time point. After a day of incubation, the RSV metabolite reached its maximum yield. The fermentation broth was extracted using a two-fold volume of MeCN, and it was vortexed, sonicated, and filtered. The filtrate was concentrated to yield the MeCN soluble fraction, which was purified by semi-preparative HPLC using a Phenomenex Luna phenyl-hexyl column (250 × 10 mm i.d., flow rate: 2 mL/min) with a solvent system containing 40% MeOH/H_2_O to yield compounds **1** (*t*_R_ 15.5 min, 2.1 mg) and **2** (*t*_R_ 34.0 min, 0.4 mg).

#### Resvebassianol A (**1**)

White amorphous powder; ${\left[\alpha \right]}_{D}^{25}$ −47.3 (*c* 0.1, MeOH); IR (KBr) *ν*_max_ 3039, 2866, 1551, 1210, 1076 cm^−1^; UV (MeOH) λ_max_ (log ε) 210 (2.4), 305 (4.0) nm; ^1^H (700 MHz) and ^13^C (175 MHz) NMR data, see Table 1; HRESIMS (negative-ion mode): *m*/*z* 403.1395 [M–H]^−^ (calcd. for C_21_H_23_O_8_, 403.1393).

### 2.5. Acid Hydrolysis of **1**

Compound **1** (1.0 mg) was hydrolyzed using 1 mL of 1N HCl under reflux conditions at 90 °C for 1 h. The hydrolysate was diluted with H_2_O, extracted using CH_2_Cl_2_ (3 × 2 mL), and the extract was evaporated in a vacuum to yield the aglycone, RSV (0.3 mg), which was identified using ^1^H NMR [13] and LC/MS analysis. The aqueous layer was neutralized by passing it through an Amberlite IRA-67 column (Rohm and Haas, Philadelphia, PA, USA), and it was repeatedly evaporated to yield the sugar unit 4-*O*-methyl-D-glucopyranose (0.4 mg, ${\left[\alpha \right]}_{D}^{25}$ +27.5 (c 0.02, MeOH)).

### 2.6. Microarray

RNA labeling and hybridization were conducted in accordance with the Agilent One-Color Microarray-Based Gene Expression Analysis protocol (Agilent Technologies, V 6.5, 2010, Lexington, MA, USA). Briefly, 200 ng of total RNA from each sample was linearly amplified, and it was labeled with Cy3-dCTP. The labeled cRNAs were purified using an RNeasy Mini Kit (Qiagen, Hilden, Germany). The concentrations and specific activities of labeled cRNAs (pmol Cy3/μg cRNA) were measured using NanoDrop ND-1000 (NanoDrop, Wilmington, NC, USA). Subsequently, 600 ng of each labeled cRNA was fragmented by adding 1 μL of 25 × fragmentation buffer and 5 μL 10 × blocking agent, and they were heated at 60 °C for 30 min. Finally, 25 μL 2 × GE hybridization buffer was added to dilute the labeled cRNA. Following this, 50 μL of hybridization solution was dispensed into the gasket slide, and it was placed in the SurePrint G3 Custom Gene Expression Microarrays, 8 × 60 K (Agilent Technologies). The slides were incubated at 65 °C for 17 h in an Agilent hybridization oven and washed at room temperature according to the Agilent One-Color Microarray-Based Gene Expression Analysis protocol (Agilent Technology, V 6.5, 2010). The hybridized array was immediately scanned using an Agilent SureScan Microarray Scanner (Agilent Technologies).

### 2.7. Cell Culture

HIEC-6, human small intestinal cells (ATCC, Manassas, VA, USA), were maintained in OptiMEM (Gibco, Waltham, MA, USA), 4% fetal bovine serum (FBS), 20 mM HEPES, 10 mM GlutaMAX, 10 ng/mL epidermal growth factor, 100 U/mL penicillin, and 100 μg/mL streptomycin. HaCaT cells, spontaneously immortalized human keratinocyte cells, were obtained from the Korean Cell Line Bank. Cells were cultured in Dulbecco’s modified eagle medium (Hyclone) supplemented with 10% FBS, 100 U/mL penicillin, and 100 μg/mL streptomycin. Subsequently, cells were incubated in a humidified atmosphere of 5% CO_2_ at 37 °C.

### 2.8. Cell Viability

To measure the cytotoxicity of compound **1** using the 3-(4,5-dimethylthiazol-2-yl)-2,5-diphenyltetrazolium bromide (MTT) assay, HIEC-6, and HaCaT cells were seeded in a 48-well plate (3 × 10^4^ cells/well) and incubated for 24 h. Subsequently, the cells were treated with different doses of resvebassianol A and RSV for 24 h. After treatment, the cells were incubated with MTT solution (0.5 mg/mL, Sigma-Aldrich, St. Louis, MO, USA) at 37 °C for 1 h. The dark blue formazan crystals were solubilized using 200 μL of DMSO per well, and the absorbance was measured at 570 nm using a spectrophotometer (Molecular Devices, San Jose, CA, USA).

### 2.9. Enzyme-Linked Immunosorbent Assay (ELISA)

Interleukin (IL)-6 and IL-1β levels were measured using ELISA. HIEC-6 and HaCaT cells were seeded (3 × 10^5^ cells/well) in a 24-well plate and stimulated using TNF-α and INF-γ (10 ng/mL each) in the presence of compound **1** and RSV. After 24 h of incubation, the supernatants were collected, and the levels of IL-1β and IL-6 were evaluated using their respective ELISA kits (R&D Systems, Minneapolis, MN, USA).

### 2.10. Cell Proliferation and Migration Assay

Cell proliferation was analyzed using the 5-Bromo-2-deoxyUridine (BrdU) assay. Briefly, HaCaT cells were seeded in a 24-well plate (4.0 × 10^4^ cells/well), treated with compound **1** or RSV for 24 h or 48 h with or without IL-22 (50 ng/mL) stimulation, and incubated with a final concentration of 10 μM BrdU. After incubation, cells were fixed at room temperature for 30 min, incubated with peroxidase-linked BrdU-antibody for 1 h, washed three times, incubated with HRP-conjugated antibody for 30 min, washed three times, and incubated with 3,3′,5,5′-tetramethylbenzidine solution for 30 min. Subsequently, the absorbance was measured at 450 nm. To measure the cell migration rates, cells were seeded at a density of 3.0 × 10^4^ cells per well in 96-well ImageLock plates (Essen Bioscience, Arbor, MI, USA) and incubated to form a spatially uniform monolayer. Scratch wounds were created in monolayers of cultured cells using a Wound Maker™ (Essen Bioscience). After creating the scratch wound, cells were washed twice with phosphate-buffered saline, incubated with or without IL-22, and treated with compound **1** or RSV in fresh serum-free media. The plate was placed in an IncuCyte^®^ ZOOM (Essen Bioscience), and the migrating cell images were recorded after 6 h of wound creation.

### 2.11. Statistical Analysis

Results of the statistical analyses are expressed as mean ± SEM. Statistical comparisons were made between the control and other groups by Bonferroni’s test for multiple comparisons of one-way analysis of variance using the GraphPad Prism 5.0 software (GraphPad Software Inc., San Diego, CA, USA). A *p*-value less than 0.05 indicated statistical significance.

## 3. Results and Discussion

### 3.1. Identification of RSV Metabolite with 4-O-Methyl-D-Glucopyranose through Biotransformation by Beauveria bassiana

**Figure 1:**
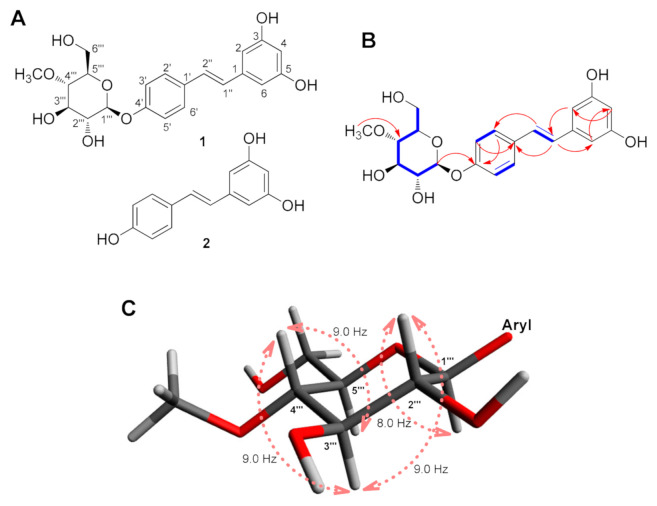
(**A**) Chemical structures of resvebassianol A (**1**) and RSV (**2**). (**B**) Key ^1^H-^1^H COSY (blue bold lines) and HMBC (red arrows) correlations of **1**. (**C**) Coupling constant analysis of 4-*O*-methyl-D-glucopyranose.

To investigate the metabolite profile of RSV obtained from biotransformation using *B. bassiana*, RSV was incubated in the culture medium with *B. bassiana* for 0, 1, 3, and 7 days. The medium samples were analyzed using HPLC, where the concentration of RSV significantly decreased with increasing incubation time, which indicates the bio-conversion of RSV (Figure S1 (Supplementary Materials)). As the biotransformation yields were monitored at each time point, the RSV metabolite reached its maximum yield after a day of incubation. Chemical analysis of the extract of the 1-day fermentation broth resulted in the isolation of a stilbene glycoside, named resvebassianol A (**1**) and RSV (Figure 1A) through semi-preparative HPLC purification.

### 3.2. Structural Elucidation of Resvebassianol A (**1**)

Resvebassianol A (**1**) was isolated as a white amorphous powder and possessed a molecular formula of C_21_H_24_O_8_ as established by the HRESIMS ion at *m*/*z* 403.1395 [M–H]^−^ (calculated for C_21_H_23_O_8_, 403.1393) and the NMR data (Table 1). The NMR data (Table 1) of **1** suggested that compound **1** shares the resveratrol skeleton by inspection and comparison of the NMR spectroscopic data with those of RSV [13], which was isolated in this study as compound **2**. In addition, the signals of oxygenated protons were observed at *δ*_H_ 3.21 (1H, t, *J* = 9.0 Hz), 3.43 (1H, ddd, *J* = 9.0, 5.0, 2.0 Hz), 3.47 (1H, dd, *J* = 9.0, 8.0 Hz), 3.57 (1H, t, *J* = 9.0 Hz), 3.71 (1H, dd, *J* = 12.0, 5.0 Hz), 3.86 (1H, dd, *J* = 12.0, 2.0 Hz), and 4.90 (1H, d, *J* = 8.0 Hz), which was deduced to be attributable to the sugar unit together with the presence of a methoxyl group at *δ*_H_ 3.59 (3H, s). With the clear evidence of compound **1** being a resveratrol glycoside, the ^13^C NMR spectrum (Table 1) also showed the signals for the resveratrol frame and the rest of the carbon NMR resonances corresponding to the sugar unit at *δ*_C_ 102.2, 80.7, 78.1, 77.3, 75.1, and 62.2, and a methoxyl group at *δ*_C_ 61.1.

The proton at *δ*_H_ 4.90 (1H, d, *J* = 8.0 Hz), attached to the downfield carbon at *δ*_C_ 102.2 (C-1′′′) with the aid of HSQC, was assigned to the anomeric proton, and its large *J*-value (8.0 Hz) was indicative of the *β*-oriented anomeric proton. A comprehensive investigation of the 2D NMR spectra of **1** allowed the establishment of its pyranose unit from C-1′′′ to C-6′′′ (Figure 1B) by ^1^H-^1^H COSY correlations from H-1′′′ to H-6′′′ and HMBC correlation from methoxyl proton (*δ*_H_ 3.59) to C-4′′′ (*δ*_C_ 80.7). Based on this evidence and comparison of the NMR data of the sugar moiety in previous reports [14], the sugar unit was determined to be 4-*O*-methyl-*β*-glucopyranose. By the analysis of vicinal coupling constants of the sugar moiety (*J*_1′′′,2′′′_ = 8.0 Hz, *J*_2′′′, 3′′′_ = 9.0 Hz, *J*_3′′′,4′′′_ = 9.0 Hz, and *J*_4′′′,5′′′_ = 9.0 Hz), it was confirmed that H-1′′′, H-2′′′, H-3′′′, H-4′′′, and H-5′′′ were all oriented in the axial positions of a pyranose ring (Figure 1C) [15]. The position of the sugar unit to the aglycone was determined by HMBC correlation between H-1′′′ and C-4′ (*δ*_C_ 158.8). The assignment of the D-configuration of the sugar unit was verified by the specific optical rotation value (${\left[\alpha \right]}_{D}^{25}$ +27.5 (c 0.02, MeOH)) of the sugar moiety obtained from the acid hydrolysate of **1**, which was comparable to the 4-*O*-methyl-D-glucopyranose previously reported, ${\left[\alpha \right]}_{D}^{20}$ +71 (*c* 0.30, MeOH) [14]. Detailed analysis of COSY and HMBC correlations confirmed the complete structure of **1** as 4′-*O*-(4′′′-*O*-methyl-*β*-D-glucopyranosyl)-resveratrol, which we named resvebassianol A.

Interestingly, resvebassianol A is a glycosylated RSV with a unique sugar unit, 4-*O*-methyl-D-glucopyranose, and it is remarkable that natural products bearing the unique sugar moiety, including akanthol, meromusides A–B, and pyridovericin-*N*-*O*-glucopyranoside, were mostly isolated from entomopathogenic fungi [16,17,18,19,20]. Recently, the glycosyltransferase–methyltransferase (GT–MT) gene pair that encodes a methylglucosylation functional module has been identified in *B. bassiana* [21]. This GT–MT gene pair is unique to the entomopathogenic fungal species, leading to the characteristic methylglycosylated natural products of these organisms [21]. The GT–MT gene pair is promiscuous in conjugating methylglucose to a broad range of drug-like substrates, but it yields both *O*- and *N*-glucosides with substantial regio- and stereoselectivity. The resulting methylglycosylated products in the recent study included the production of resvebassianol A [21], yet the absolute configuration was established for the first time in the present study.

### 3.3. Microarray Analysis to Determine the Functional Differences between Resvebassianol A and RSV

**Figure 2:**
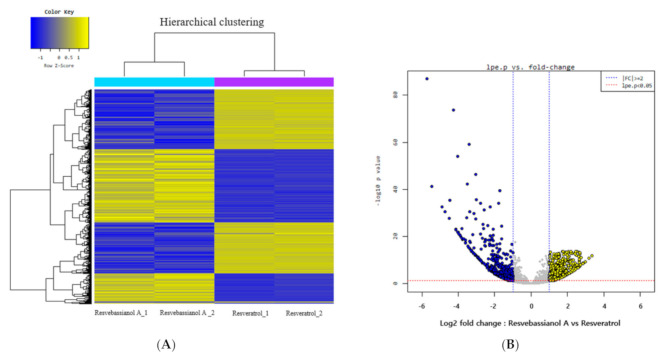
Differential expression of genes in resvebassianol A- and RSV-treated keratinocytes. (**A**) Hierarchical clustering of altered mRNA. Microarray analysis for mRNA expression patterns of platelet heatmap of deregulated mRNAs, which were two-fold upregulated or downregulated. (**B**) Volcano plotting microarray analysis revealed the mRNAs that were two-fold upregulated or downregulated in platelet during storage.

We investigated the gene expression profiles of resvebassianol A- and RSV-treated skin keratinocytes using microarray analysis to determine the functional differences between resvebassianol A and RSV. Analyses of the gene expression data of both groups revealed 1790 differentially expressed genes with a fold-change higher than two. It was observed that 843 genes were upregulated, while 947 genes were downregulated in resvebassianol A-treated cells (Figure 2). The expression of several genes, including six upregulated genes and eight downregulated genes, was analyzed (Table 2).

**Figure 3:**
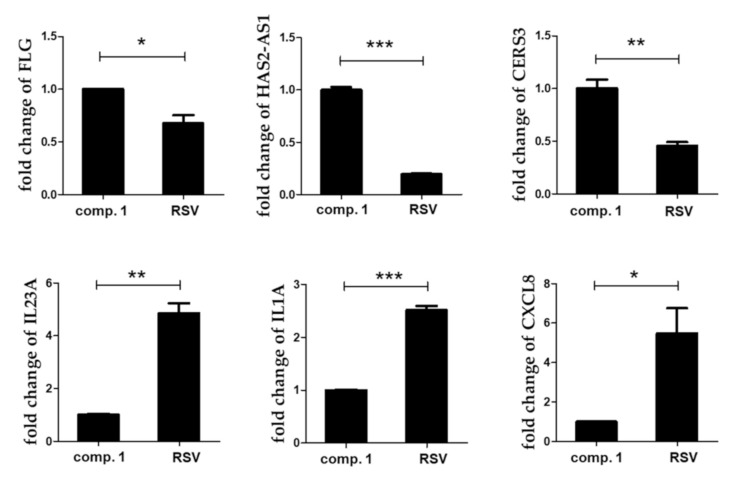
The key differentially expressed mRNAs identified from microarray were verified using qRT-PCR. The expression of genes in resvebassianol A (**1**)- and RSV-treated groups was consistent with the results of gene chip detection. Values are expressed as means ± SD. * *p* < 0.05, ** *p* < 0.01, and *** *p* < 0.001 vs. RSV-treated group; Comp. means compound.

To further validate the results of microarray analyses, qRT-PCR was performed to confirm the expression of differentially expressed genes. The expression levels of many selected genes (FLG, HAS2-AS1, CERS3, IL23A, IL1A, and CXCL8) were examined via qRT-PCR using the same RNA samples. The expression levels of filaggrin (FLG), HAS2-AS1, and CERS3 were higher, while those of IL23A, IL1A, and CXCL8 were lower in the resvebassianol A-treated group than in the RSV-treated group. Thus, the results of qRT-PCR were consistent with those of the microarray analysis (Figure 3). The functions of these altered genes were mainly related to the maintenance of skin barrier function, immune reaction, and cell regeneration of keratinocytes derived from the skin epidermis.

### 3.4. Effects of Resvebassianol A on the Proliferation and Migration of HaCaT Cells

**Figure 4:**
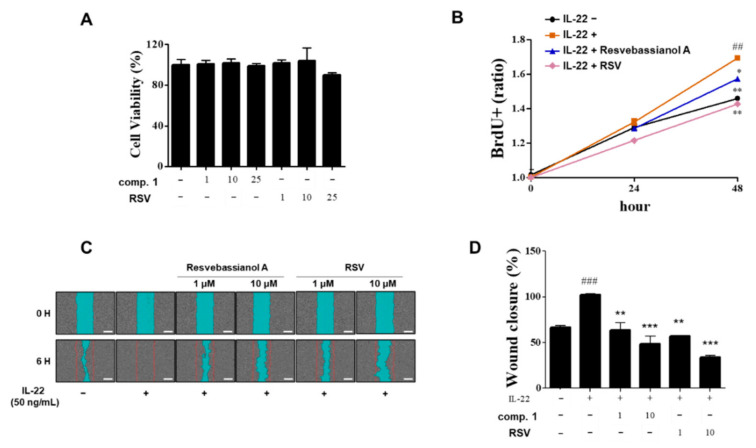
Effects of resvebassianol A on the proliferation and migration of HaCaT cells. (**A**) Cells were cultured in 96-well plates, and they were treated with resvebassianol A and RSV (1, 10, and 25 μM). After 24 h cell viability was measured using the MTT assay. (**B**) HaCaT cell proliferation after 24 and 48 h of treatment with resvebassianol A and RSV was measured using BrdU incorporation assay. (**C**) The wound margin was photographed after 0 h and 6 h of wound scratching. (**D**) Quantitative analysis of wound closure was determined as the wound area at a given time relative to that of the IL-22-treated group. Values are expressed as means ± SEM. ^##^ *p <* 0.01 and ^###^ *p* < 0.001 versus untreated (control) group; * *p* < 0.05, ** *p* < 0.01, and *** *p* < 0.001 vs. IL-22-treated group.

Next, the proliferation effects of resvebassianol A were tested in HaCaT cells using the BrdU assay (Figure 4). Interestingly, resvebassianol A showed no cytotoxicity, whereas RSV showed cytotoxicity at 25 μM (Figure 4A). At the cellular and animal model levels, most glycosidic compounds are safer and have higher stability than aglycones. After comparing the proliferation rates of RSV and resvebassianol A, it was observed that the latter significantly inhibited cell proliferation from 24 h to 48 h, which was similar to the activity of RSV in IL-22-induced keratinocytes without any cytotoxicity (Figure 4B). To investigate the potential effects of resvebassianol A on cell migration and rejuvenation in skin keratinocytes, we performed a cell scratch assay and wound analysis. The wound areas in IL-22-induced HaCaT cells were measured after a 6 h treatment with resvebassianol A or RSV (1, 10 μM each, Figure 4C). Resvebassianol A inhibited cell migration; however, the same concentration of RSV showed higher inhibition (Figure 4C,D) in IL-22-induced HaCaT cells. The difference in proliferation and migration rates indicated that resvebassianol A showed effects similar to those of RSV without any cytotoxicity.

### 3.5. Inhibitory Effects of Resvebassianol A on the Inflammatory Cytokine Expression of TNF-α/INF-γ-Induced HIEC-6 Cells

**Figure 5:**
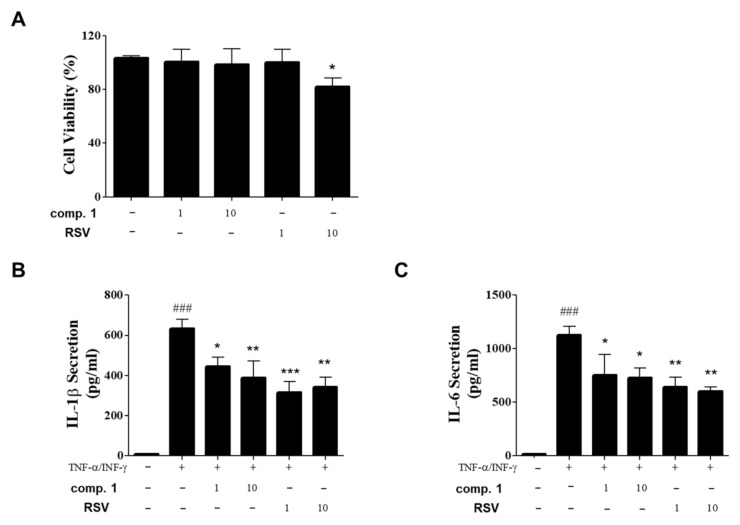
Inhibitory effects of resvebassianol A on the inflammatory cytokine expression of TNF-α/INF-γ-induced HIEC-6 cells. (**A**) Cells were cultured in 96-well plates, and they were treated with resvebassianol A and RSV at 1 and 10 μM, respectively. After 24 h, cell viability was measured using the MTT assay. (**B**,**C**) The levels of IL-6 and IL-1β in the supernatants were determined using ELISA. Values are expressed as means ± SD. ^###^ *p* < 0.001 versus control group; * *p* < 0.05, ** *p* < 0.01, and *** *p* < 0.001 vs. TNF-α/IFN-γ-treated group.

To confirm the regenerative and anti-inflammatory effects of resvebassianol A in other tissues as well as in skin cells, we examined the inhibitory effects of resvebassianol A on inflammatory cytokine secretion in TNF-α/IFN-γ-stimulated HIEC-6 cells and human intestinal epithelial cells. Treatment with resvebassianol A showed no cell death, in contrast to RSV treatment (Figure 5A). Resvebassianol A significantly inhibited both IL-6 and IL-1β secretion in a dose-dependent manner, but the anti-inflammatory activity of RSV was higher than that of resvebassianol A (Figure 5B,C). These results suggest the inhibition of inflammatory cytokine secretion by resvebassianol A was similar to that of RSV with no cytotoxicity. Thus, resvebassianol A may maintain epithelial homeostasis and repair the damage caused by various toxic factors in skin and intestinal tissues. Based on these findings, resvebassianol A was determined to be safe for use even at high concentrations, unlike RSV, which is cytotoxic at high concentrations.

RSV has various biological activities in humans [22]. Particularly, many studies have reported the effectiveness of RSV in skin photoaging, inflammation, skin whitening, and skin cancer [23,24,25,26]. Despite its various biological functions, RSV has very low structural stability and bioavailability upon oral administration [27]. Additionally, it is known to interact with other drugs [28]. We have conducted several studies to investigate the application of RSV, its synthetic derivatives, and RSV-enriched rice as functional cosmeceuticals [23,29], where RSV showed cytotoxicity in skin cells at high concentrations. Therefore, we conducted this study to establish a new method for producing RSV derivatives using biotransformation on the entomopathogenic fungus *B. bassiana*, which led to the biosynthesis of an interesting RSV derivative with enhanced stability and safety. Glycosylation is widespread among natural products and is capable of increasing the diversity of structure and function of natural products [30]. Glycosylation of natural products can be advantageous in terms of water solubility, pharmacological activities, pharmacokinetic properties, and bioavailability [30], which provides useful information for the development and application of glycosylated natural products for drug research and development.

Recently, attempts were made to use the characteristic methylglycosylated natural products from *B. bassiana* for biosynthesis, where the resulting methylglycosylated products showed increased solubility and displayed increased stability against glycoside hydrolysis [21]. Upon methylglucosidation, specific methylglycosylated products were found to exhibit enhanced bioactivities [21]. These findings support our results establishing a new method for the biosynthesis of methylglycosylated RSV with enhanced safety and stability using biotransformation by *B. bassiana*.

## 4. Conclusions

In this study, we suggest a practical method for the efficient biosynthesis of a wide range of natural glycosides in total biosynthetic or biocatalytic platforms. Despite many trials for the development of a drug using RSV, there have been limitations associated with the metabolism and bioavailability of RSV. Our findings introduce the method for the production of a unique RSV metabolite, resvebassianol A, which is less toxic and has higher stability upon oral administration compared to RSV, while exhibiting the biological functions that RSV possesses. Until now, most studies on biotransformation have been intensively conducted on endogenous interactions using endophytes; instead, we used edible *B. bassiana* possessing excellent pharmacological activities in the skin and other diseases [20,21,31]. The current study will establish a foothold in successfully overcoming the problems in the development of RSV-derived drugs, while introducing an economically and ecologically friendly method for producing the RSV derivatives.

## Figures and Tables

**Table 1:** ^1^H (700 MHz) and ^13^C NMR (175 MHz) Data of Resvebassianol A (**1**) in CD_3_OD ^a^.

Position	*δ*_H_	*δ*_C_
1		141.0
2	6.46 d (2.0)	106.0
3		159.8
4	6.18 t (2.0)	103.1
5		159.8
6	6.46 d (2.0)	106.0
1′		133.2
2′	7.45 d (8.5)	128.7
3′	7.07 d (8.5)	118.0
4′		158.8
5′	7.07 d (8.5)	118.0
6′	7.45 d (8.5)	128.7
1″	6.88 d (16.0)	128.7
2″	6.99 d (16.0)	129.1
1′′′	4.90 d (8.0)	102.2
2′′′	3.47 dd (9.0, 8.0)	75.1
3′′′	3.57 t (9.0)	78.1
4′′′	3.21 t (9.0)	80.7
5′′′	3.43 ddd (9.0, 5.0, 2.0)	77.3
6′′′	3.71 dd (12.0, 5.0); 3.86 dd (12.0, 2.0)	62.2
4′′′-OCH_3_	3.59 s	61.1

^a^ Signal multiplicity is expressed as doublet (d), doublet of doublet (dd), doublet of doublet of doublet (ddd), and triplet (t) and coupling constants (Hz) are in parentheses.

**Table 2:** Upregulated or downregulated genes in resvebassianol A-treated keratinocytes compared to those in RSV-treated cells.

Gene Symbol	RefSeq	Gene Name	Fold-Change
CCL17	NM_002987	Chemokine (C-C motif) ligand 17	4.79
FLG	NM_002016	Filaggrin	4.02
HAS2-AS1	NR_002835	HAS2 antisense RNA 1	3.43
IL17RE	NM_153483	Interleukin 17 receptor E	3.13
TLR3	NM_003265	Toll-like receptor 3	3.00
CERS3	NM_178842	Ceramide synthase 3	2.62
IL11RA	NM_001142784	Interleukin 11 receptor, alpha	−2.24
IL7R	NM_002185	Interleukin 7 receptor	−2.62
IL32	NM_001012631	Interleukin 32	−3.53
IL1A	NM_000575	Interleukin 1, alpha	−3.66
CXCL8	NM_000584	Chemokine (C-X-C motif) ligand 8	−3.90
IL23A	NM_016584	Interleukin 23, alpha subunit p19	−7.53
IL4I1	NM_152899	Interleukin 4 induced 1	−9.30
IL11	NM_000641	Interleukin 11	−13.16

## Supplementary Materials

The following are available online at https://www.mdpi.com/article/10.3390/biomedicines9050555/s1, Figure S1: HPLC analysis of RSV metabolites obtained from biotransformation of RSV by *B. bassiana*; Figure S2: The HR-ESIMS data of compound **1**; Figure S3: The ^1^H NMR spectrum of compound **1** (CD_3_OD, 700 MHz); Figure S4: The ^13^C NMR spectrum of compound **1** (CD_3_OD, 175 MHz); Figure S5: The ^1^H-^1^H COSY spectrum of compound **1**; Figure S6: The HSQC spectrum of compound **1**; Figure S7: The HMBC spectrum of compound **1**.
