# Supplementary material for: Generation of Stilbene Glycoside with Promising Cell Rejuvenation Activity through Biotransformation by the Entomopathogenic Fungus Beauveria bassiana
Source: Biomedicines. 2021 May 17;9(5):555. doi: 10.3390/biomedicines9050555 (PMC8156121; doi:10.3390/biomedicines9050555)
Supplement: Supplementary file 1 [file biomedicines-09-00555-s001.zip › biomedicines-1203568-SI.pdf]

Supporting Information

Generation of Stilbene Glycoside with Promising Cell Rejuvenation Activity through Biotransformation by the Entomopathogenic Fungus *Beauveria bassiana*

Sang Keun Ha [1,2,†], Min Cheol Kang [3,†], Seulah Lee [4,5,†], Om Darlami [3], Dongyun Shin [3], Inwook Choi [1,*], Ki Hyun Kim [4,*], and Sun Yeou Kim [3,*]

[1]Division of Functional Food Research, Korea Food Research Institute, Sungnam, Gyeonggi-do 463-746, Republic of Korea

[2]Divison of Food Biotechnology, University of Science and Technology, Daejeon, Republic of Korea

[3]College of Pharmacy, Gachon University, #191, Hambakmoero, Yeonsu-gu, Incheon 21936, Republic of Korea

[4]School of Pharmacy, Sungkyunkwan University, Suwon 16419, Republic of Korea

[5]Division of Life Sciences, Korea Polar Research Institute, KIOST, Incheon, 21990, Republic of Korea

Supporting Information Contents:

Figure S1. HPLC analysis of RSV metabolites obtained from biotransformation of RSV by *B. bassiana*…………………………………....S3
Figure S2. The HR-ESIMS data of compound **1**……………..……………………..……...………………………………………………..S4
Figure S3. The ^{1}H NMR spectrum of compound **1** (CD_3OD, 700 MHz)……………………………...………………..……………………S5
Figure S4. The ^{13}C NMR spectrum of compound **1** (CD_3OD, 175 MHz)……………………………...………………..…………………...S6
Figure S5. The ^{1}H-^{1}H COSY spectrum of compound **1**……………………………...……………..………………………………………...S7
Figure S6. The HSQC spectrum of compound **1**……………………………...…………..……………………………………………….....S8
Figure S7. The HMBC spectrum of compound **1**……………………….………………………………………………………………………S9
General Experimental Procedures ..……………..………………………….………………………………………………………………...…S10

Figure S1. HPLC analysis of RSV metabolites obtained from biotransformation of RSV by *B. bassiana*

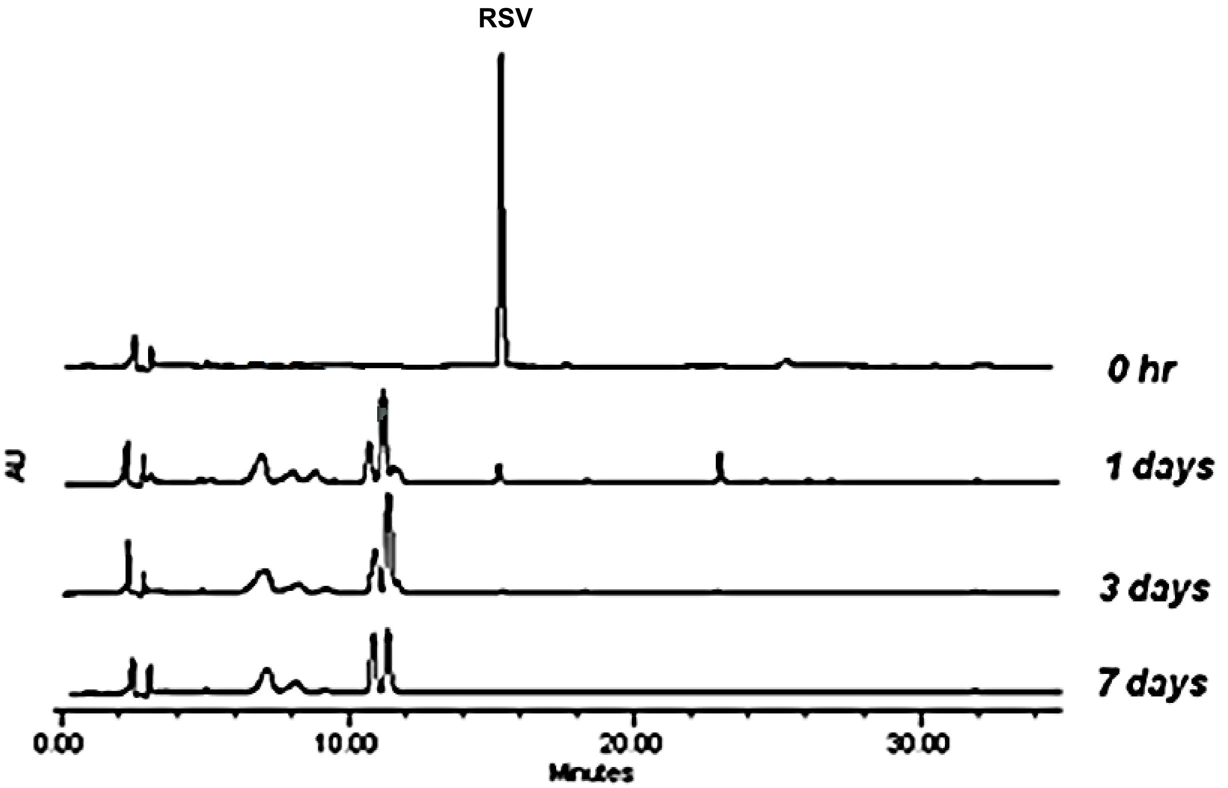

* Chromatographic data were monitored at 305 nm.

Figure S2. The HR-ESIMS data of compound **1**

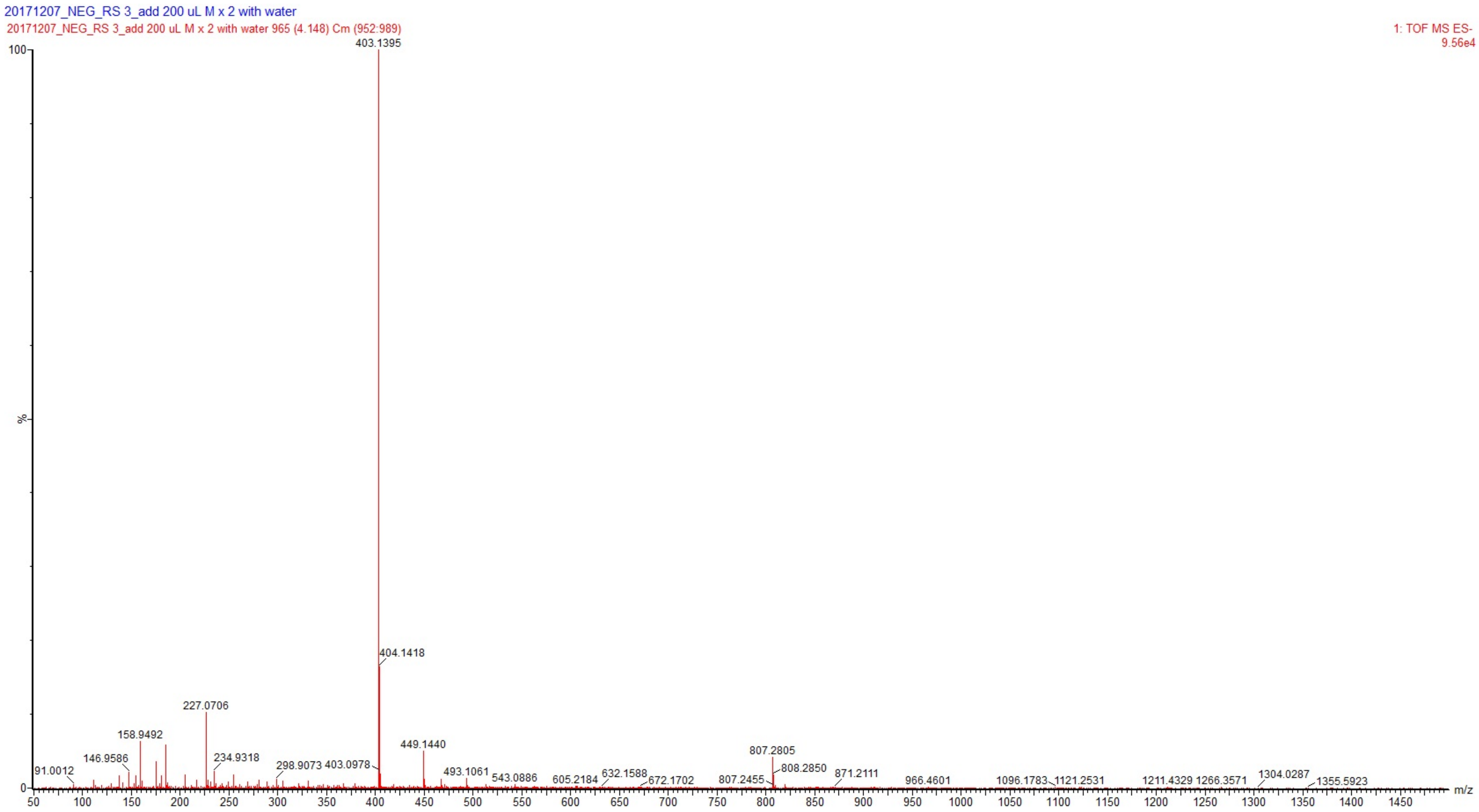

Figure S3. The ^{1}H NMR spectrum of compound **1** (CD_3OD, 700 MHz)

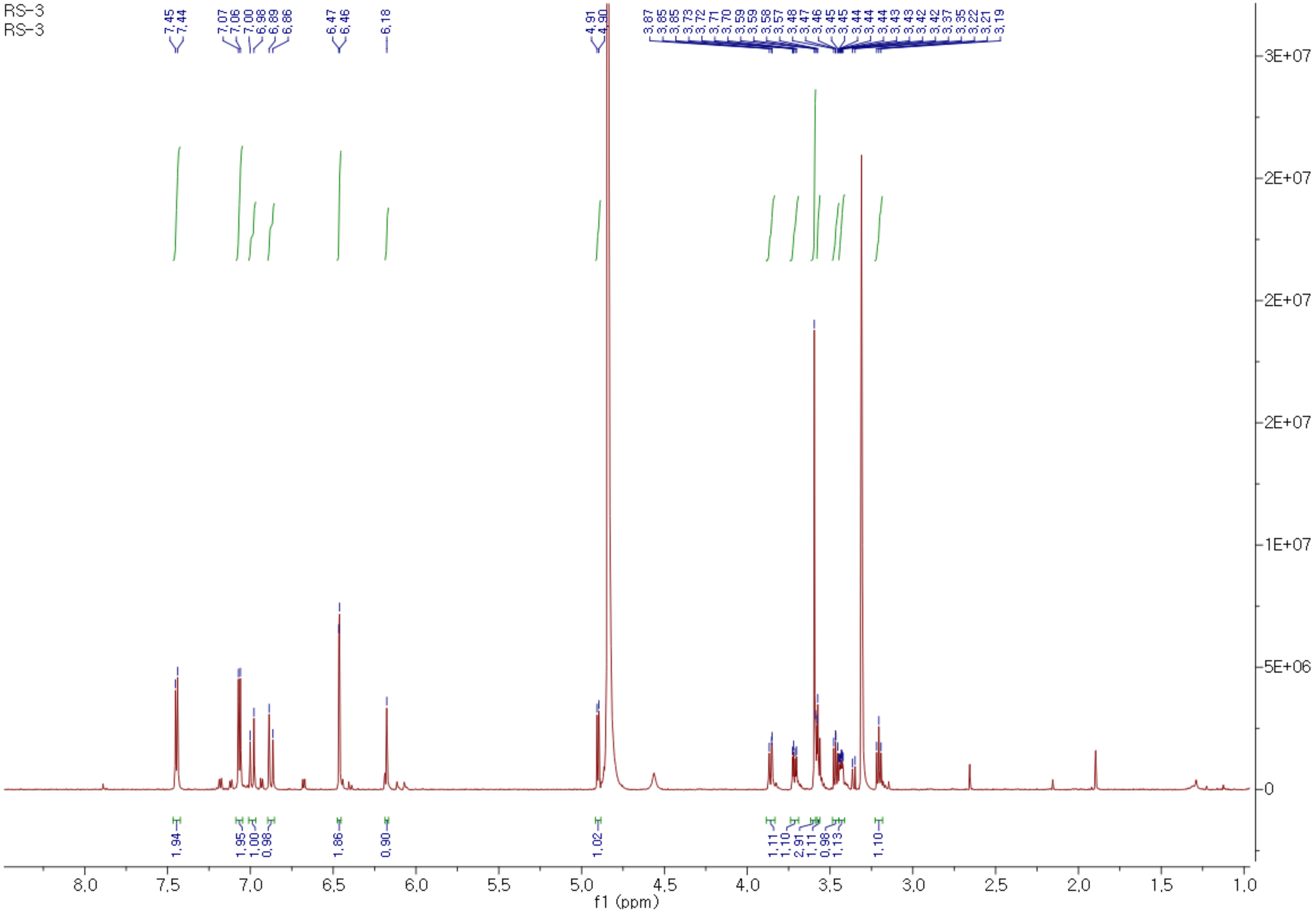

Figure S4. The ^{13}C NMR spectrum of compound **1** (CD_3OD, 175 MHz)

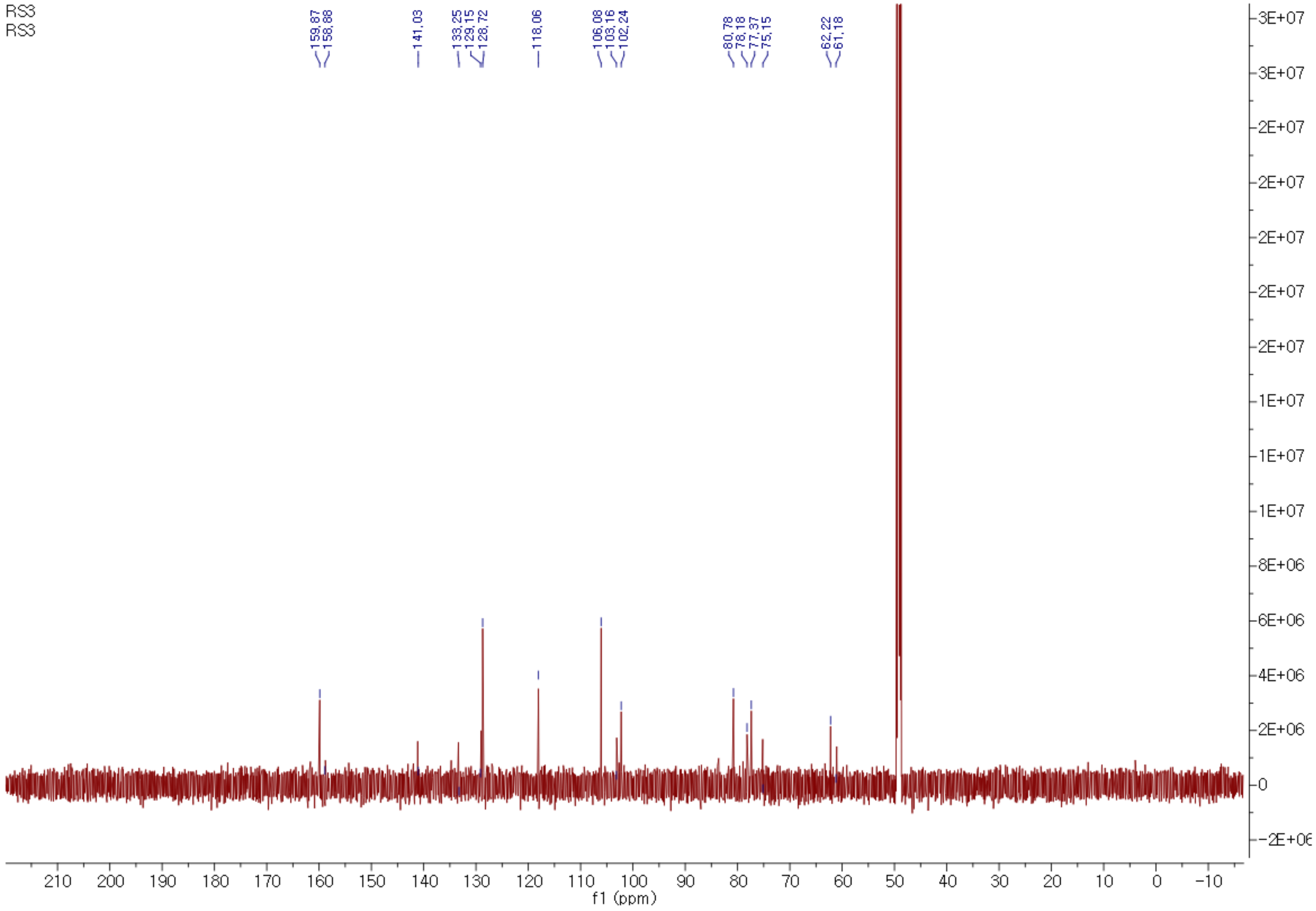

Figure S5. The ^{1}H-^{1}H COSY spectrum of compound **1**

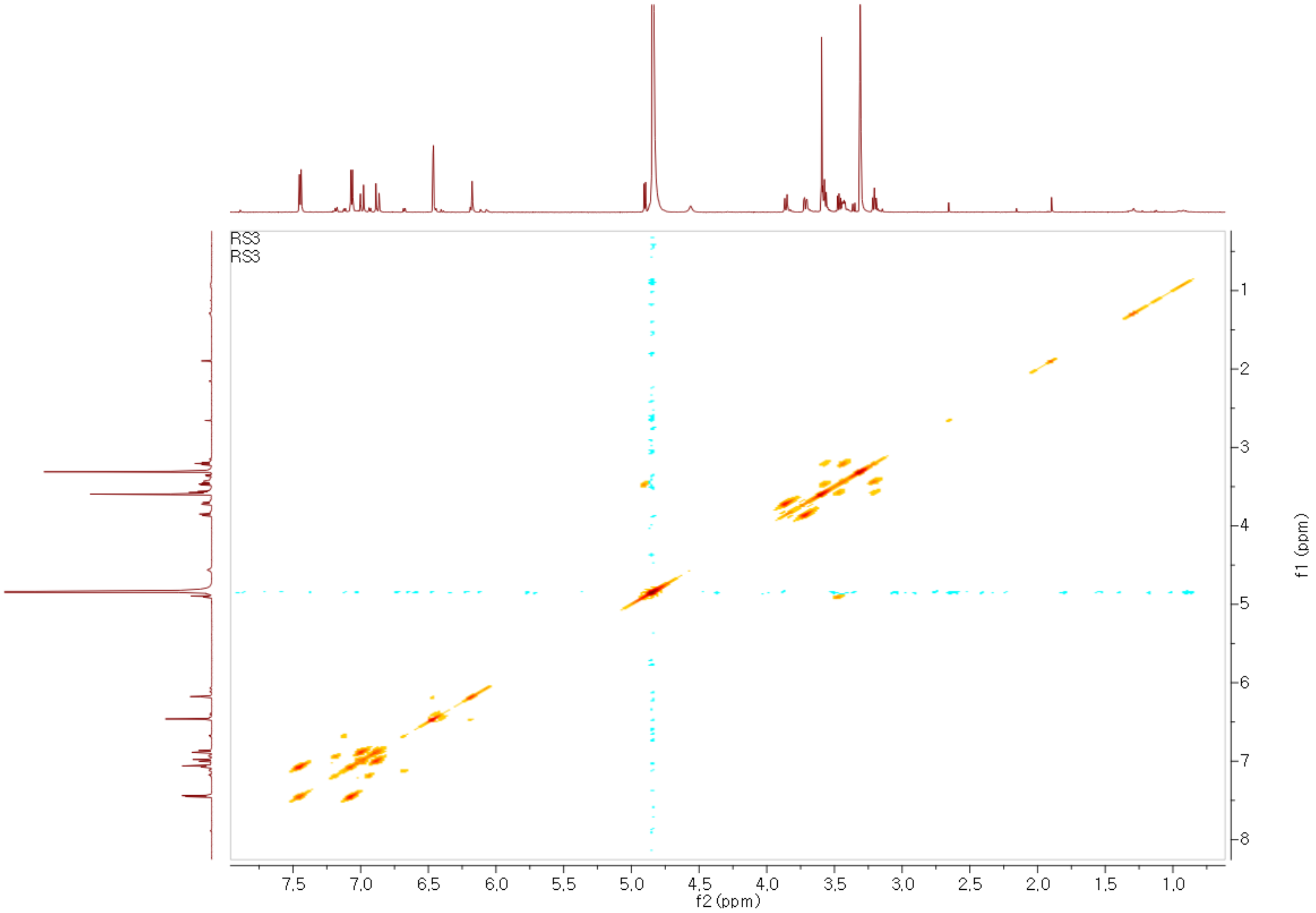

Figure S6. The HSQC spectrum of compound **1**

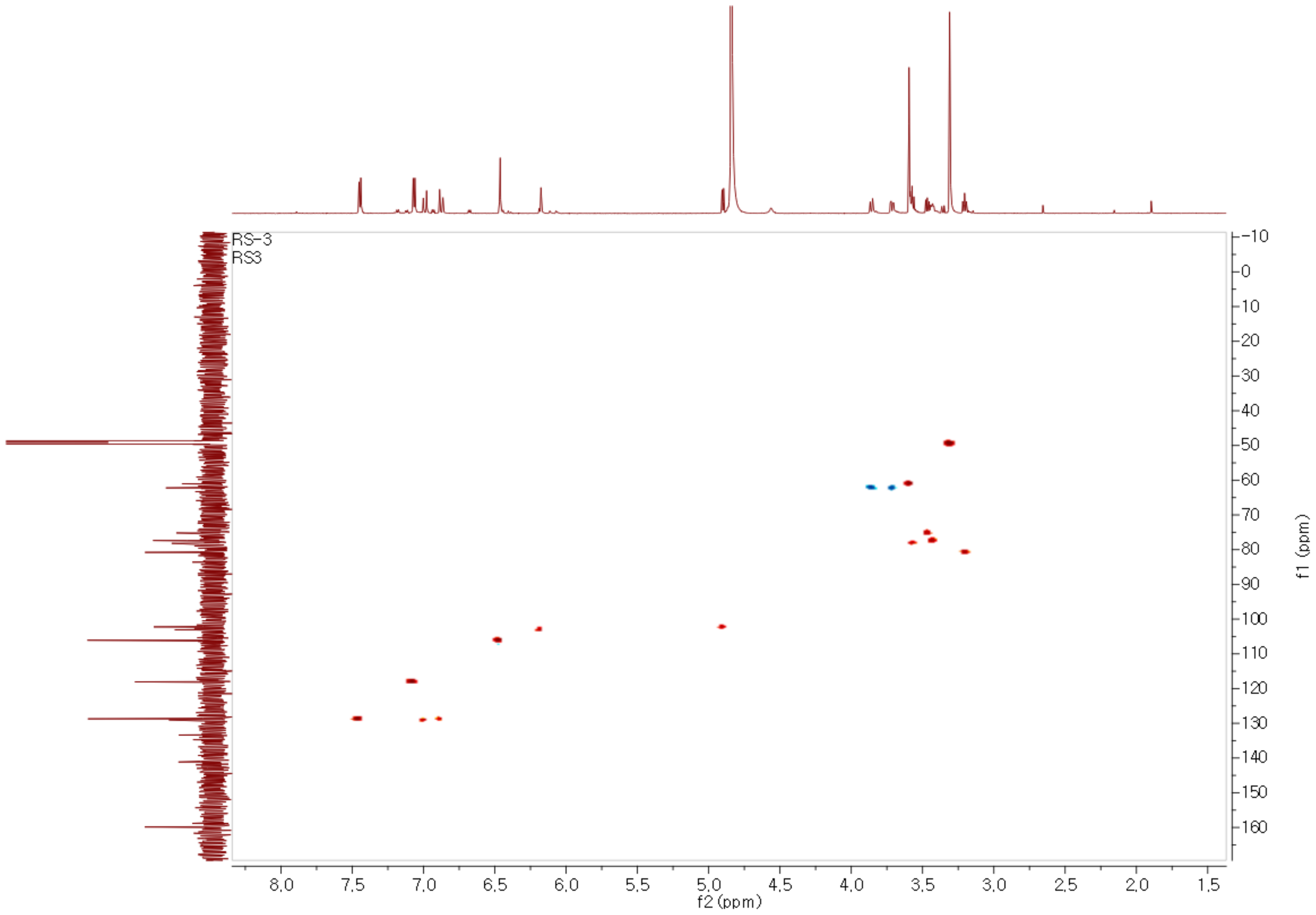

Figure S7. The HMBC spectrum of compound **1**

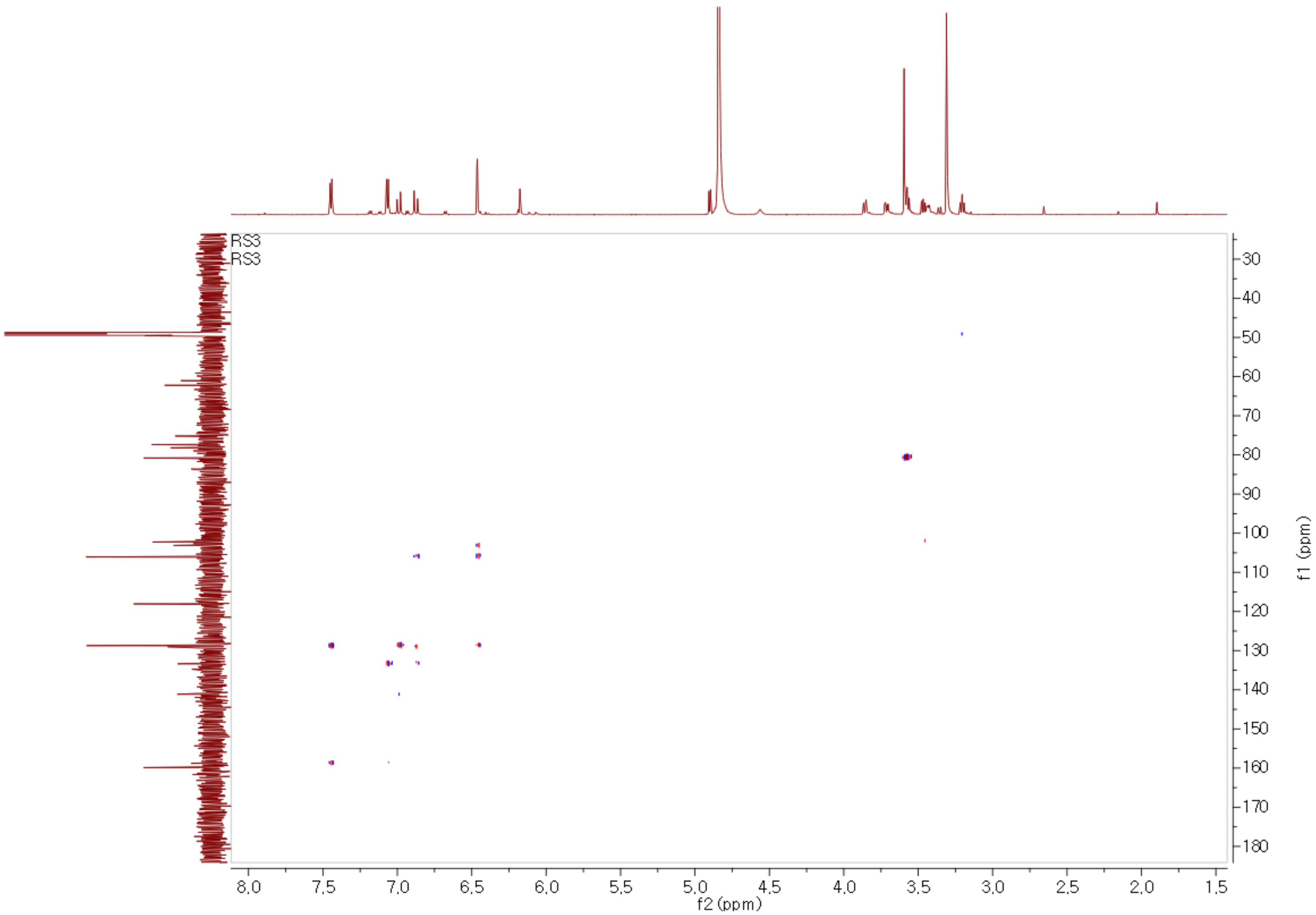

General Experimental Procedures

Optical rotations were measured using a Jasco P-2000 polarimeter (Jasco, Easton, MD, USA). Infrared (IR) spectra were acquired on an IFS-66/s FT-IR spectrometer (Bruker, Karlsruhe, Germany). Ultraviolet (UV) spectra were acquired on an Agilent 8453 UV-visible spectrophotometer (Agilent Technologies, Santa Clara, CA, USA). Nuclear magnetic resonance (NMR) spectra were recorded using a Bruker AVANCE III NMR spectrometer operating at 700 MHz (^1H) and 175 MHz (^{13}C) (Bruker). Semi-preparative HPLC was performed using a Shimadzu Prominence HPLC System with SPD-20A/20AV Series Prominence HPLC UV-Vis detectors (Shimadzu, Tokyo, Japan) and a Phenomenex Luna Phenyl-Hexyl column (250 × 10 mm, 10 μm; flow rate: 2 mL/min; Phenomenex, Torrance, CA, USA). LC/MS analysis was performed on an Agilent 1200 Series HPLC system equipped with a diode array detector and 6130 Series ESI mass spectrometer using an analytical Kinetex C_{18} 100 Å column (100 × 2.1 mm, 5 μm; flow rate: 0.3 mL/min; Phenomenex). HRESIMS data were obtained using a Waters Xevo G2 QTOF mass spectrometer and Synapt G2 HDMS quadrupole time-of-flight (TOF) mass spectrometer (Waters Corp., Milford, CT, USA). Thin-layer chromatography (TLC) was performed using precoated silica gel F_{254} plates and RP-C_{18} F_{254s} plates (Merck); the spots were detected under UV light or by heating after spraying with anisaldehyde-sulfuric acid. Standard resveratrol (RSV) was purchased from Sigma (MO, USA), and HPLC grade acetonitrile (MeCN) and water were purchased from J.T. Baker (NJ, USA). American Chemical Society grade chemicals were used in the study.
